# Corynebacterium amycolatum peritonitis in a patient undergoing peritoneal dialysis: case report and literature review

**DOI:** 10.1099/acmi.0.000880.v3

**Published:** 2024-10-30

**Authors:** Fatima Zahra Adil, Imane Aragon, Elmostafa Benaissa, Yassine Ben Lahlou, Fatna Bssaibis, Adil Maleb, Mariama Chadli, Mostafa Elouennass

**Affiliations:** 1 Bacteriology Department, Mohammed V Military Teaching Hospital, Rabat, Morocco; 2 Nephrology Department, Mohammed V Military Teaching Hospital, Rabat, Morocco; 3 Research Team of Epidemiology and Bacterial Resistance, Faculty of Medicine and Pharmacy, Rabat, Morocco

**Keywords:** *Corynebacterium amycolatum*, peritoneal dialysis, peritonitis

## Abstract

Peritoneal dialysis is a blood purification technique used in cases of end-stage chronic kidney failure, based on the filtering capabilities of the peritoneum. Infections, often caused by poor asepsis during catheter manipulation, are generally attributed to *Staphylococcus epidermidis* and *Staphylococcus aureus. Corynebacterium*, usually considered non-pathogenic, is rarely involved in these infections. We present a case of peritonitis due to *Corynebacterium amycolatum* in a patient undergoing peritoneal dialysis. The diagnosis was made based on cytobacteriological examination of the dialysate fluid, which on two occasions showed high levels of white blood cells with a predominance of neutrophilic polymorphonuclear and a monomorphic appearance of colonies on agar medium, whose identification by biochemical tests and antibiotic sensitivity study confirmed the presence of *C. amycolatum*. The patient was successfully treated with vancomycin, resulting in symptom resolution and sterilization of the dialysate fluid. Although rare, the involvement of *Corynebacterium* species underscores the importance of confirming its pathogenicity. Further studies are needed to better understand the epidemiology of these infections and guide future treatments. This case also highlights the need for a rigorous approach to confirming the pathogenicity of *Corynebacterium* despite its traditional classification as a contaminant.

## Data Summary

No data were yielded by this research, and its replication does not require any.

## Introduction

Peritoneal dialysis is an extrarenal treatment used for end-stage chronic renal failure, leveraging the peritoneum’s filtration abilities. Through a catheter, it facilitates exchanges between blood and dialysate [1]. However, mishandling and failure to adhere to aseptic protocols often lead to catheter-related infections, the primary cause of peritonitis in peritoneal dialysis. This negligence permits skin microbiota bacteria to infiltrate the peritoneal cavity, contributing to the majority of peritonitis cases [2,3]. The most frequently involved pathogens in this type of infection are *Staphylococcus epidermidis* and *Staphylococcus aureus* [3]. The genus *Corynebacterium* is part of the normal skin microbiota. It has rarely been associated with cases of peritonitis in patients undergoing peritoneal dialysis [4]. *Corynebacterium amycolatum* is a Gram-positive, non-motile bacterium, typically presenting as short rods with irregular shapes, often aligning in V-shaped patterns or palisade formations. It is catalase-positive and grows well on standard media such as blood agar, exhibiting gamma haemolysis. *C. amycolatum* possesses nitrate reductase, enabling it to reduce nitrate to nitrite, but it does not ferment glucose. This species is notable for its natural resistance to several antibiotics, including penicillin [5].

We report the first case in Morocco of peritonitis in peritoneal dialysis caused by *C. amycolatum*.

## Case presentation

A 47-year-old patient with end-stage chronic renal failure who had been started on peritoneal dialysis was brought to the emergency department of Mohamed V Military Teaching Hospital complaining of abdominal pain. Despite being afebrile during the clinical examination, the dialysis fluid appeared cloudy, prompting a sample to be sent to the microbiology laboratory of the same facility for cytobacteriological examination.

**Fig. 1.**
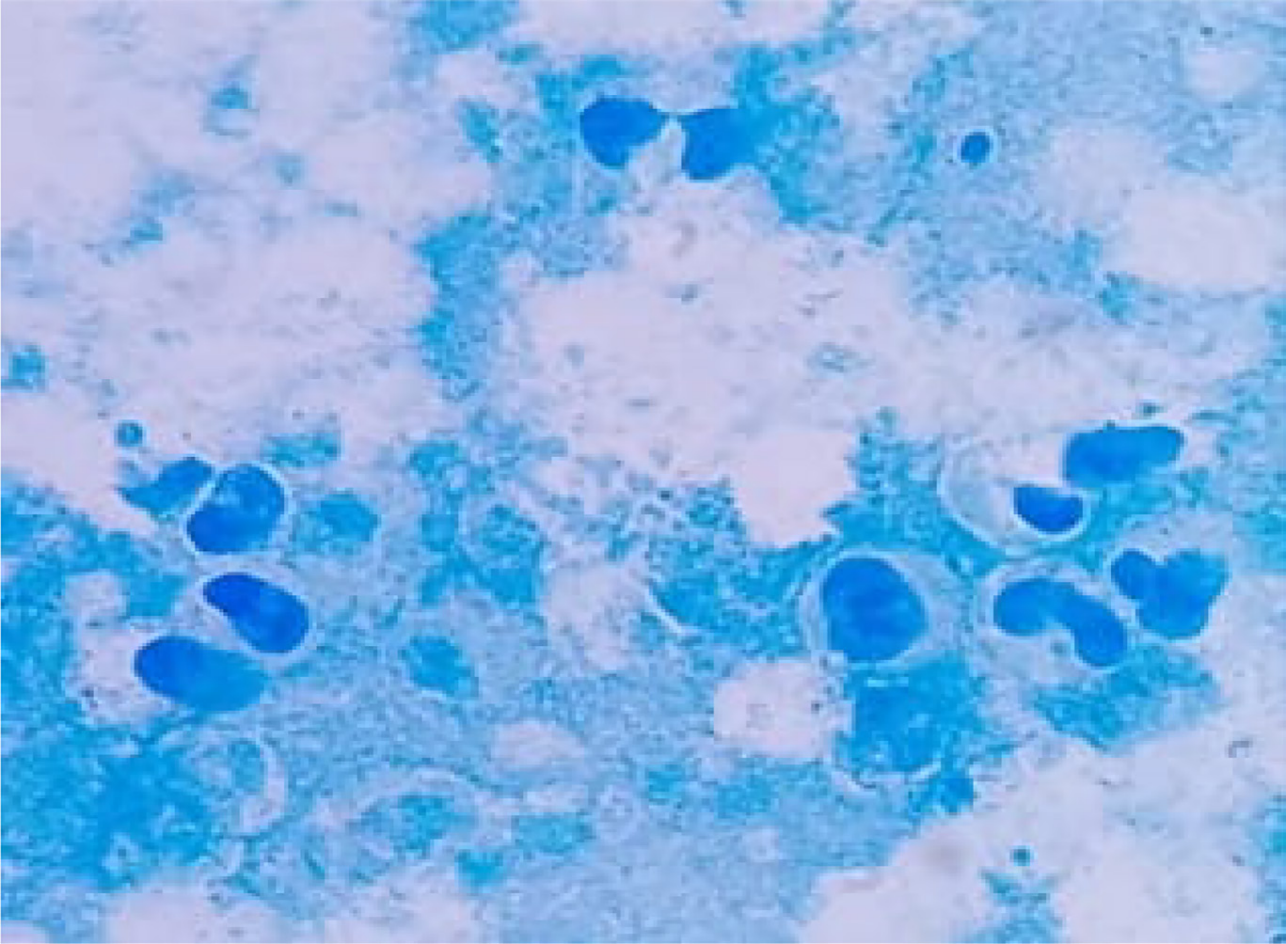
Neutrophilic polymorphonuclear cells on a smear stained with methylene blue.

The specimen was cultured across various media, including blood agar, enriched chocolate agar, blood agar supplemented with nalidixic acid and colistin and Schaedler agar. Incubation of the first two mediums occurred at 37 °C under aerobic conditions with CO_2_, whereas the latter two were subjected to anaerobic incubation. The cytological examination was performed using Kova Slide counting cells, revealing white blood cell and red blood cell counts of 670 mm^−3^ and 32 mm^−3^, respectively. The leukocyte formula showed a predominance of neutrophilic polymorphonuclear cells at 98%, while lymphocytes were at 2% (Fig. 1).

**Fig. 2.**
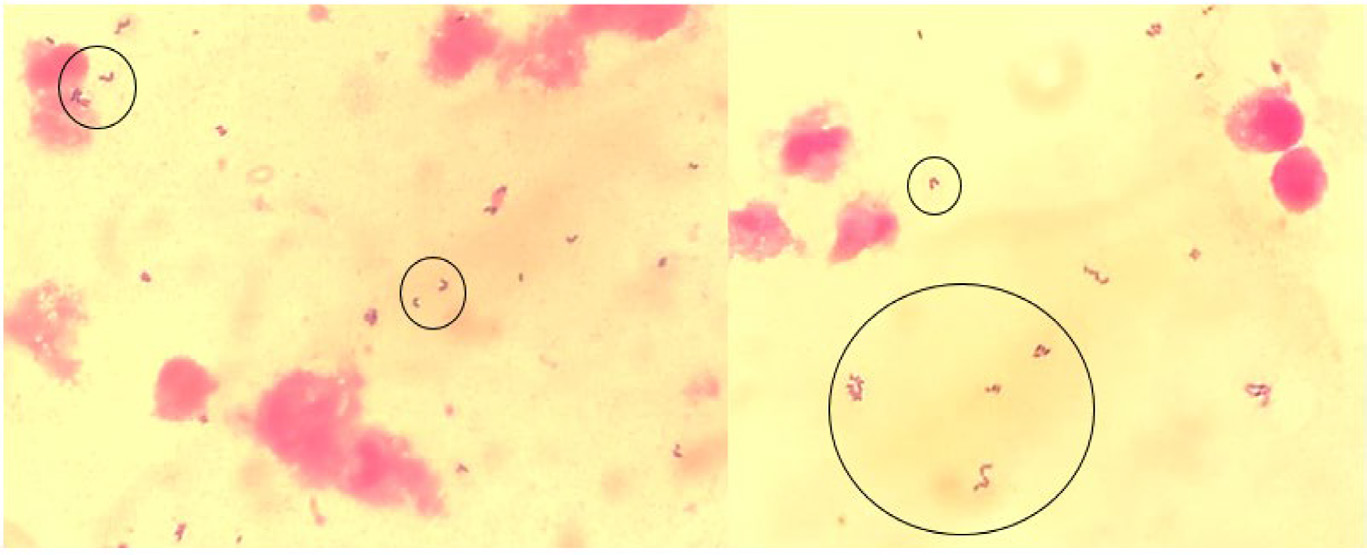
Gram-positive bacilli on a Gram-stained smear.

Microscopic examination after Gram staining revealed the presence of Gram-positive bacilli (Fig. 2).

**Fig. 3.**
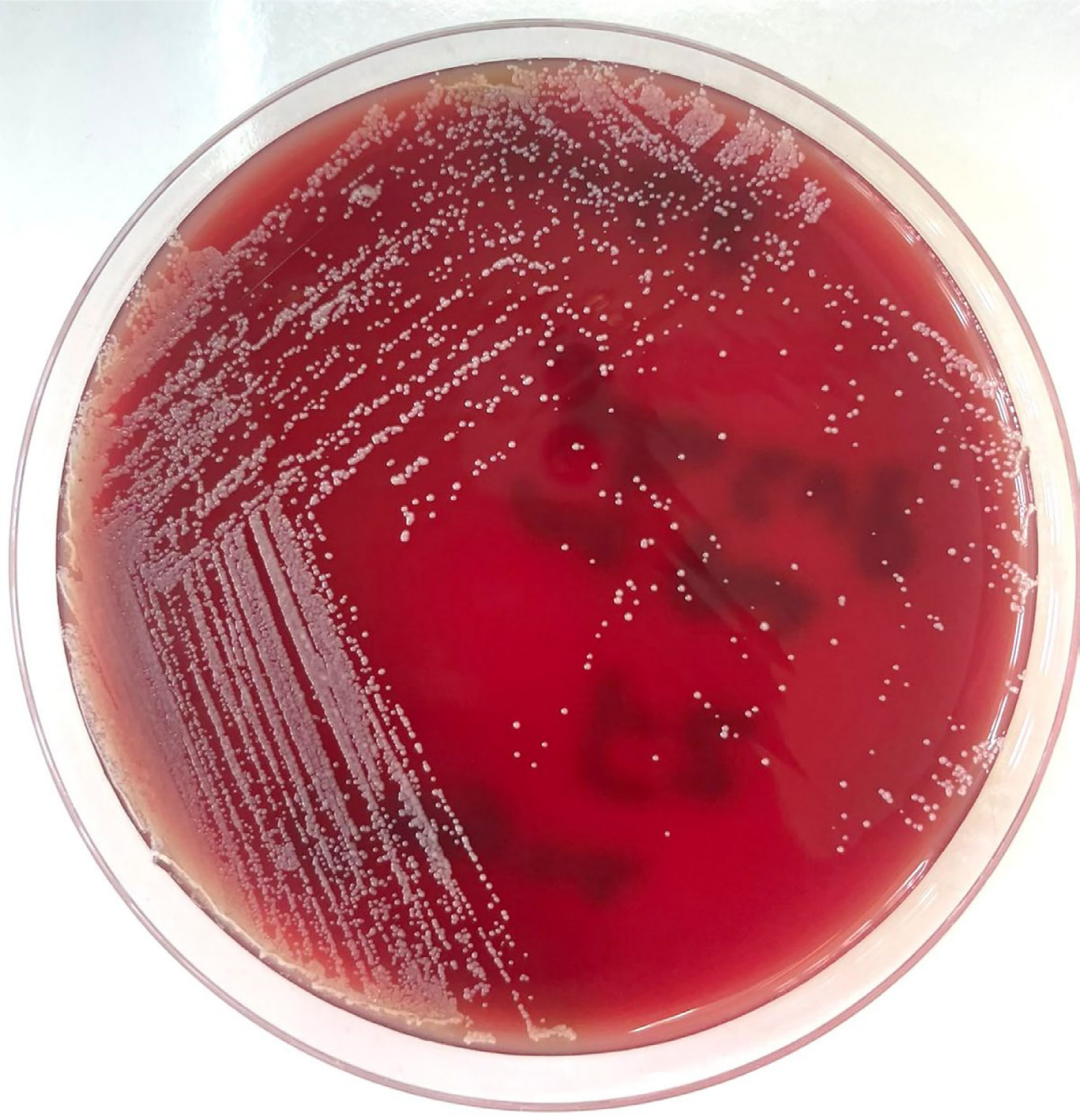
Colonies of *C. amycolatum* on blood agar.

After incubating the aerobic cultures for 24 h and the anaerobic cultures for 48 h, all agar media showed colonies with a monomorphic appearance of Gram-positive coryneform bacilli (Fig. 3).

Species identification was performed by analysing biochemical characteristics using an API Coryne gallery (bio-Mérieux SA, Marcy l’Étoile, France) and by matrix-assisted laser desorption ionization – time of flight, yielding *C. amycolatum* with a probability of 99 and 99.9%, respectively. Antimicrobial susceptibility testing was carried out using the disc diffusion method, following the guidelines of the Antibiotic Susceptibility Committee of the French Society of Microbiology and the European Committee on Antimicrobial Susceptibility Testing [6]. The obtained results are represented in Table 1.

**Table 1.** Results of antimicrobial susceptibility testing

Antibiotic	Breakpoint (mm)		Obtained inhibition zone (mm)	Result
	S ≥	R <		
Penicillin G	29	29	**6**	**R**
Ciprofloxacin	50	25	**6**	**R**
Linezolid	25	25	36	S
Rifampicin	30	30	38	S
Tetracycline	24	24	31	S
Trimethoprim/sulfamethoxazole	19	16	**6**	**R**
Vancomycin	17	17	19	S

S: Susceptible, R: Resistant.

Moreover, the sample was further enriched in an aerobic blood culture bottle (BD BACTEC Plus medium), yielding positive results consistent with the initial culture. Non-specific biological markers indicated a C-reactive protein level of 17.4 mg l^−1^ (normal range <5 mg l^−1^), with all other assessments within normal limits. To exclude alternative infectious causes, cultures on Lowenstein Jensen medium and smears stained with Ziehl–Neelsen stain to detect acid-fast bacilli were conducted. Furthermore, PCR GeneXpert (Xpert MTB/RIF) was employed to investigate the presence of the *Mycobacterium tuberculosis* complex, all yielding negative results. The mycological examination also returned negative findings.

To confirm the pathogenicity of *C. amycolatum* in this case, a second sample was sent to the laboratory 4 days later. It was handled using the same steps as the first sample. Cytological examination showed white blood cell and red blood cell counts of 1550 mm^−3^ and 55 mm^−3^, respectively. The leukocyte formula indicated a predominance of neutrophilic polymorphonuclear cells at 99%, with lymphocytes at 1%. The results of Gram staining, culture and antibiogram, as well as enrichment on an aerobic blood culture bottle, were identical to the first sample.

Following these results, the patient received 1 g of vancomycin through intraperitoneal administration every 5 days for a duration of 3 weeks. The antibiotic choice was guided by the antibiogram, which confirmed bacterial sensitivity to vancomycin, to avoid the risk of therapeutic failure associated with cephalosporins. Additionally, the dialysis catheter remained unchanged. Post-treatment, he reported no further abdominal pain, and the dialysis fluid cleared. A subsequent cytobacteriological examination after 3 weeks revealed a decrease in the white blood cell count (160 mm^−3^) and a sterile culture. The same results were observed after 6 months.

## Discussion

Peritoneal dialysis accounts for 11% of dialysis techniques used and 9% of renal replacement therapies worldwide [7]. Peritonitis, characterized by the infection of peritoneal fluid and inflammation of the peritoneum, has a mortality rate of 18% among all infectious causes and is the leading reason for switching to haemodialysis. Dialysate contamination occurs mainly through the endoluminal route and less frequently from an intra-abdominal infection site or an infection at the catheter’s exit site [8]. The most common pathogens are coagulase-negative staphylococci, especially *S. epidermidis*, which frequently colonizes the skin and hands, as well as *S. aureus*. Less commonly, when peritonitis arises from an intra-abdominal source, *Enterobacterales*, *Streptococcus* sp. and anaerobic bacteria are involved [3].

The genus *Corynebacterium* includes numerous species of Gram-positive bacilli that are part of the skin and mucosal microbiota, alongside *Staphylococcus* sp. and *Micrococcus* sp. [3,4]. These bacteria are generally viewed as having low pathogenicity [3]. In clinical samples, *Corynebacterium*, once dismissed as contamination, is now acknowledged as a pathogen capable of causing peritonitis. Consequently, the management of *Corynebacterium* peritonitis has been incorporated into the guidelines established by the International Society for Peritoneal Dialysis (ISPD) since 2010 [9]. The involvement of the *Corynebacterium* genus in peritonitis during peritoneal dialysis does not exceed 5% [3]. A study conducted in Australia and New Zealand among patients undergoing peritoneal dialysis reported a *Corynebacterium* peritonitis rate of 1.5% [2], while in the study by Barraclough *et al*., the rate was 2.3% [4].

To date, five cases of peritonitis on peritoneal dialysis due to *C. amycolatum* have been reported, including three adults [10,12] and two children [13]. All were cases of recurrent peritonitis, but this was not the case in our instance. Identifying species is crucial in a clinical context, as it helps differentiate between chronic and newly acquired infections. This distinction allows for the appropriate adjustment of antibiotic therapy and may lead to the decision to remove a peritoneal dialysis catheter if the same strain is repeatedly isolated [13].

Regarding therapeutic management, the choice of antibiotic remains a subject of ongoing discussion. One study indicated that the use of cefazolin was associated with favourable outcomes [4], while another revealed a recurrence rate of 48% with the same antibiotic, preferring vancomycin for better results [14]. However, a study conducted by Htay *et al*. found that patients treated with cefazolin had outcomes similar to those treated with vancomycin [2]. The treatment duration recommended by the ISPD is 3 weeks [9].

## Conclusion

The involvement of *Corynebacterium* species in peritonitis occurring during peritoneal dialysis, although rare, requires special attention. The traditional classification of *Corynebacterium* as a contaminant underscores the importance of thorough confirmation of its pathogenicity in these cases. Additional studies are essential to deepen our understanding of the bacterial epidemiology of these infections and to guide future therapeutic decisions.

## Abbreviation

ISPD: International Society for Peritoneal Dialysis
